# Primary Unilateral Ethmoidal Ewing Sarcoma With Orbital Extension: A Report of a Rare Case

**DOI:** 10.7759/cureus.105541

**Published:** 2026-03-20

**Authors:** Vedavyas Muvva, Inuganti Venkata Renuka, Vaddatti Tejeswini, Chaitra B, Priyanka Kumbha

**Affiliations:** 1 Pathology, NRI Medical College, Chinnakakani, IND

**Keywords:** case report, ewing sarcoma, oncology, pathology, sinonasal mass

## Abstract

Ewing Sarcoma (EWS) is a rare, aggressive, small round blue cell tumor that predominantly arises in children and young adults. Sinonasal localization is a rare and difficult presentation to diagnose due to nonspecific clinical and histopathological features.

A 21-year-old male patient presented with acute left-sided epistaxis associated with periorbital swelling and epiphora. Contrast-enhanced CT revealed a well-defined lobulated heterogeneously enhancing iso-hyperdense lesion in the left ethmoid sinus with extensions and mass effect. Histopathological examination demonstrated features of a small round blue cell tumor. Immunohistochemistry showed diffuse nuclear positivity for NKX2.2 and focal membranous positivity for CD99, with negative staining for pan-cytokeratin, CD45, chromogranin, and synaptophysin, confirming the diagnosis of EWS.

The patient was counseled regarding multimodal treatment options, including surgery, chemotherapy, and radiotherapy. However, he declined treatment and was discharged against medical advice, and was subsequently lost to follow-up.

Sinonasal EWS is a rare entity with overlapping histomorphological features that complicate the diagnosis. A systematic approach incorporating histopathology and immunohistochemistry is essential for accurate identification. This case highlights an extremely rare unilateral ethmoid sinus presentation of sinonasal EWS and underscores the importance of considering it in the differential diagnosis of sinonasal masses in young adults.

## Introduction

Ewing sarcoma (EWS) is a rare, aggressive, small round blue cell tumor that predominantly arises in children and young adults. Small blue round cell tumors are a group of aggressive malignant tumors with hyperchromatic nuclei and scant cytoplasm. The estimated incidence of EWS is approximately one case per 1.5 million individuals [1]. Although EWS can arise in any part of the body, it most commonly involves the long bones of the limbs and the pelvis. Approximately 20% of cases are extra-osseous in origin [1]. In 1% to 4% of the cases of EWS, the head and neck region is the primary site of the tumor [2]. Within this region, sinonasal localization is particularly rare; hence, only 17 cases have been reported in the literature to our knowledge. The rarity of this presentation often leads to diagnostic challenges, as the clinical and radiological features may overlap with other more common sinonasal pathologies.

We report a case of a 21-year-old male patient with primary EWS of the left ethmoid sinus, which is an extremely rare presentation of this malignancy. Through this case, we aim to highlight the clinical and histopathological features of sinonasal EWS and emphasize the importance of considering it in the differential diagnosis of a sinonasal mass in young adults.

## Case presentation

**Figure 1:**
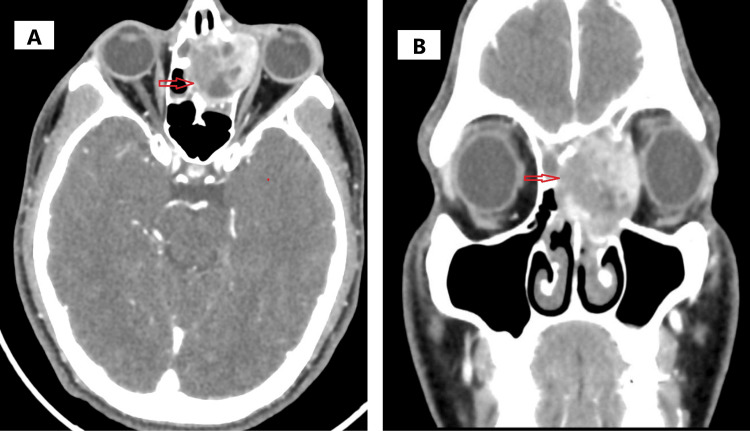
Radiology images A&B: Contrast-enhanced CT scan showing a well-defined lobulated lesion in the left ethmoid sinus with orbital extension.

A 21-year-old male patient with a history of hypertension, who was not on any antihypertensive or other medications, presented with left-sided epistaxis for one day. The bleeding was associated with left eye swelling and epiphora. He complained of bouts of sneezing and reported having had recurrent upper respiratory tract infections. He also complained of paroxysmal nocturnal dyspnea. The patient denied any trauma. On physical examination of the nose, the osseocartilaginous framework was intact, and the nasal pyramid was symmetrical. On tip raise, nares and vestibule were normal. Anterior rhinoscopy revealed mild deviation of the nasal septum to the right, bilateral inferior turbinate hypertrophy on the lateral walls, and blood clots were seen on the left lateral wall. On the cold spatula test, there was equal misting on both sides. On the cotton wool test, equal movement was seen on both sides. A contrast-enhanced CT of the paranasal sinuses was done, which reported a well-defined lobulated heterogeneously enhancing iso-hyperdense lesion in the left ethmoid sinus with orbital extensions and mass effect (Figures 1A, 1B).

Diagnostic nasal endoscopy and biopsy of the mass were done after a week, during which an assessment of the hypertension was done, which was unrelated to the epistaxis. The laboratory hematological findings were within normal limits.

**Figure 2:**
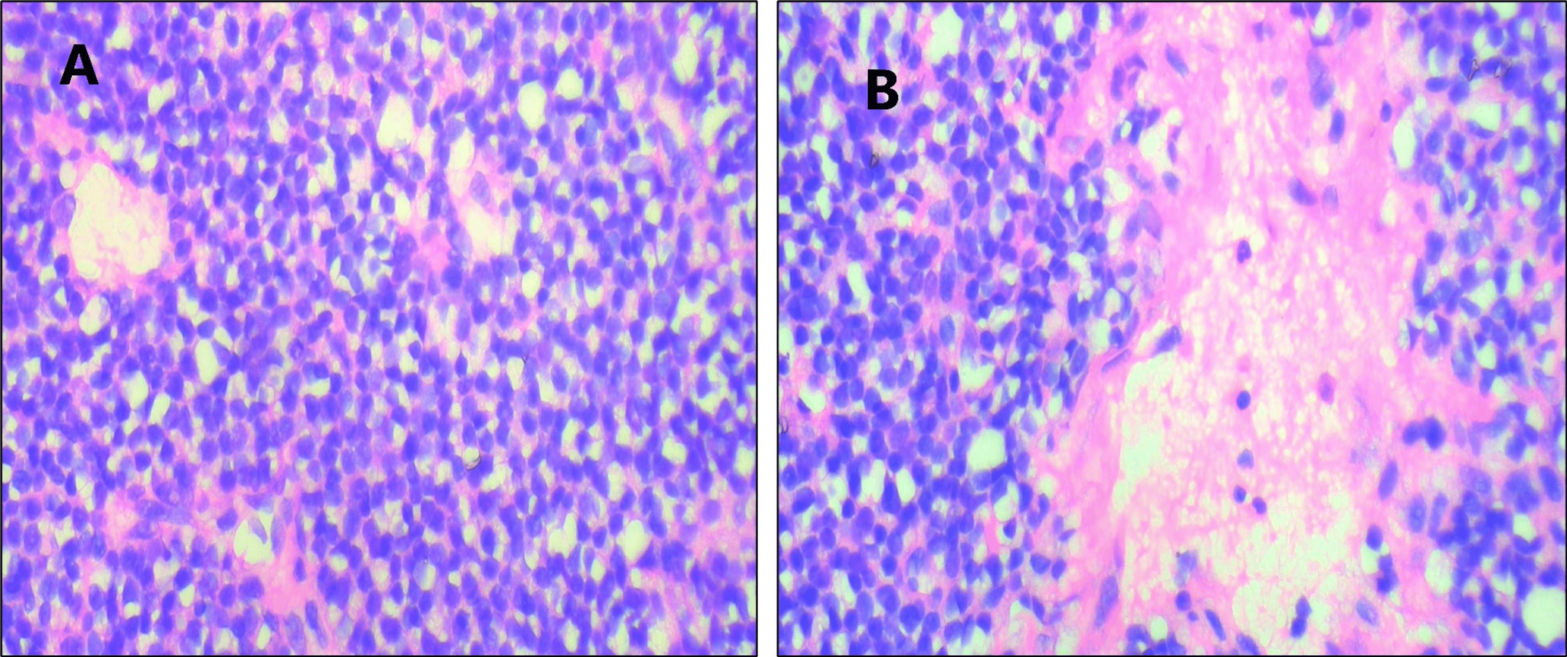
Microphotographs of the tumor A) Microphotograph of tumor with sheets and nests of monotonous round cells with hyperchromatic nuclei and moderate cytoplasm (H&E, 400x); B) Cells with moderate eosinophilic cytoplasm and inconspicuous nucleoli. The tumor cells are interspersed with multiple dilated vascular channels and areas of necrosis (H&E, 400X).

Grossly, multiple gray-white tissue bits altogether measuring 0.6 x 0.6 cm were seen. All the tissue was embedded. Microscopy revealed fragments of mucosa lined by pseudostratified ciliated columnar epithelium with mucus glands. There were sheets and nests of monotonous round cells with hyperchromatic nuclei, moderately eosinophilic to clear cytoplasm, and inconspicuous nucleoli. The tumor cells were infiltrated by multiple dilated vascular channels (Figures 2A, 2B).

**Figure 3:**
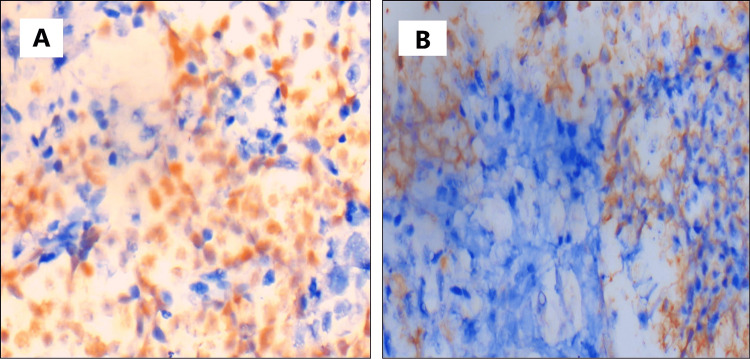
Immunohistochemistry (IHC) images A) Microphotograph showing NKX 2.2 diffuse nuclear positivity in tumor cells (IHC 400x); B) Microphotograph showing CD99 focal membranous positivity in tumor cells (IHC x 400x)

The mitotic activity was indiscernible. There were areas of necrosis and hemorrhage. This histological appearance was consistent with a small round blue cell tumor. Given the site of the lesion and the histological appearance, the various differential diagnoses considered were sinonasal neuroblastoma, sinonasal undifferentiated carcinoma (SNUC), small cell neuroendocrine carcinoma (SNEC), EWS, and lymphoma. To narrow the diagnosis, we proceeded to do immunohistochemistry testing (Table 1; Figures 3A, 3B)

**Table 1:** Summary of immunohistochemistry (IHC) markers

IHC Marker	Result
NKX2.2	Diffuse positive
CD99	Focal positive
Pan-CK	Negative
CD45	Negative
Chromogranin	Negative
Synaptophysin	Negative

The final diagnosis was reported as extraosseous paranasal EWS.

Following the diagnosis of EWS, we had discussions with the patient where we educated him on his condition. We had explained the various treatment modalities available to him. The patient opted not to undergo any treatment. 

## Discussion

Shaari et al. conducted a review of the literature, reviewing 93 reported cases of sinonasal EWS [3]. In their study, they reported that sinonasal EWS presents with nonspecific clinical findings, including nasal obstruction, epistaxis, and impaired vision [3]. Our patient’s presentation of acute left-sided epistaxis accompanied by left periorbital swelling and epiphora is consistent with these findings. The orbital extension and mass effect explain the patient's left eye swelling. In their study, they had reported a mean age of diagnosis as 26.4 years (range one to 89 years), while our case was diagnosed at the age of 21 years [3]. Additionally, a male predominance (53%) was observed in their study, which aligns with the demographic profile of our patient [3]. Sinonasal EWS was most commonly found in the maxillary sinus, accounting for 35% of cases [3]. Left-sided involvement was reported in 44 cases (47%), while bilateral disease was observed in only four cases (4%) [3]. Lin and Liang reported a 26-year-old man presenting with persistent left-sided nasal obstruction. Endoscopy demonstrated a friable mass in the left nasal cavity originating from the middle turbinate with extension into the nasopharynx [4]. In contrast, our case was unilateral and involved the left ethmoid sinus, representing a rarer pattern of presentation.

While the histologic origin of EWS is debated, it is believed that both neuroectodermal cells and mesenchymal progenitor cells are the cells of origin. In the present case, histology revealed sheets and nests of monotonous round cells with hyperchromatic nuclei, moderate eosinophilic to clear cytoplasm, and inconspicuous nucleoli, consistent with a small round blue cell tumor. Small round blue cell tumors of the sinonasal region pose a significant diagnostic challenge due to their overlapping histomorphological features and broad differential diagnoses. Simons et al. provided a systematic approach for pathologists to aid in the diagnosis of sinonasal small round blue cell tumors and recommended immunohistochemistry testing if histologic findings are nonspecific [5]. In line with this approach, we had narrowed down the differentials based on histology to sinonasal neuroblastoma, SNUC, SNEC, EWS, and lymphoma. We proceeded to the immunohistochemistry to further narrow the differentials. The tumor demonstrated diffuse nuclear positivity for NKX2.2 and focal membranous positivity for CD99, while lacking expression of pan-cytokeratin, CD45, chromogranin, and synaptophysin. The absence of pan-CK expression excludes carcinomas such as SNEC and SNUC.

Additionally, the lack of chromogranin and synaptophysin expression argues against sinonasal neuroblastoma. Lymphoma is typically characterized by CD45 positivity, which was absent in this case, thereby excluding this diagnosis. The immunoprofile observed in this case, particularly the characteristic NKX2.2 positivity, strongly supports a diagnosis of EWS.

The widely accepted treatment is surgical excision with neoadjuvant or adjuvant chemotherapy, if negative margins are attainable [6]. Radiotherapy, along with chemotherapy, is the preferred treatment modality if negative margins are unattainable [6,7]. Shaari et al. reported overall survival to be 60% by this treatment approach [3]. A recent international consensus statement by Vinciguerra et al. concurs with the above approach for treatment [8]. In our case, the patient refused to undergo treatment and requested to be discharged against medical advice. Unfortunately, the patient was subsequently lost to follow-up.

Gupta et al. state that the prognosis depends on the site of the primary tumor, the presence of distant metastasis at presentation, and the age of the patient. Researchers have found that patients younger than 15 years of age and patients with axial and sinonasal tract involvement have a better prognosis [9]. 

## Conclusions

Sinonasal EWS is a rare and diagnostically challenging clinical pathology, given the nonspecific clinical features and the overlap in histomorphology with other differentials of small round blue cell tumors. This case highlights a rarer presentation, involving the unilateral left ethmoid sinus, of an already rare entity, further emphasizing the variability in the disease pattern of sinonasal EWS. A systemic approach for accurate diagnosis would be histological evaluation followed by immunohistochemistry testing. Early diagnosis and multidisciplinary interventions are essential for optimal outcomes; however, treatment decisions may be limited by patient factors, as illustrated by treatment denial and loss to follow-up in this case. Reporting such rare presentations contributes to the existing literature and aids in creating awareness in clinicians and pathologists of sinonasal EWS, and aids in improving diagnostic accuracy.
